# Molecular Quantum Spintronics: Supramolecular Spin Valves Based on Single-Molecule Magnets and Carbon Nanotubes

**DOI:** 10.3390/ijms12106656

**Published:** 2011-10-10

**Authors:** Matias Urdampilleta, Ngoc-Viet Nguyen, Jean-Pierre Cleuziou, Svetlana Klyatskaya, Mario Ruben, Wolfgang Wernsdorfer

**Affiliations:** 1 Institut Néel, CNRS et Université Joseph Fourier, BP 166, F-38042 Grenoble Cedex 9, France; E-Mails: matias.urdampilleta@grenoble.cnrs.fr (M.U.); ngoc-viet.nguyen@grenoble.cnrs.fr (N.-V.N.); jean-pierre.cleuziou@grenoble.cnrs.fr (J.-P.C.); 2 Institute of Nanotechnology (INT), Karlsruhe Institute of Technology (KIT), 76344 Eggenstein-Leopoldshafen, Germany; E-Mails: svetlana.klyatskaya@kit.edu (S.K.); mario.ruben@kit.edu (M.R.); 3 Institute de Physique et Chimie de Matériaux de Strasbourg (IPCMS), CNRS-Université de Strasbourg, 67034 Strasbourg, France

**Keywords:** molecular quantum spintronics, molecular magnets, nanoelectronics devices

## Abstract

We built new hybrid devices consisting of chemical vapor deposition (CVD) grown carbon nanotube (CNT) transistors, decorated with TbPc_2_ (Pc = phthalocyanine) rare-earth based single-molecule magnets (SMMs). The drafting was achieved by tailoring supramolecular π-π interactions between CNTs and SMMs. The magnetoresistance hysteresis loop measurements revealed steep steps, which we can relate to the magnetization reversal of individual SMMs. Indeed, we established that the electronic transport properties of these devices depend strongly on the relative magnetization orientations of the grafted SMMs. The SMMs are playing the role of localized spin polarizer and analyzer on the CNT electronic conducting channel. As a result, we measured magneto-resistance ratios up to several hundred percent. We used this spin valve effect to confirm the strong uniaxial anisotropy and the superparamagnetic blocking temperature (*T*_B_ ~ 1 K) of isolated TbPc_2_ SMMs. For the first time, the strength of exchange interaction between the different SMMs of the molecular spin valve geometry could be determined. Our results introduce a new design for operable molecular spintronic devices using the quantum effects of individual SMMs.

## 1. Introduction

Single molecule magnets (SMMs) have attracted much interest over the last years because of their unique magnetic properties. These molecular structures combine the classical properties of magnets with the intrinsic quantum nature of nanoscale entities. With a large spin ground state and a magnetic anisotropy well-defined, molecular clusters composed of few magnetic atoms have shown various properties such as the blocking of the spin orientation at low temperatures, quantum tunneling of magnetization (QTM) [1] and interference effects between tunneling paths [2]. Besides, the synthetic chemistry produces controlled molecular structures at high yield and low cost. As a result, a wide range of SMMs systems incorporating transition metal and/or rare earth metal ions with tailored magnetic interactions have been discovered. In addition, the rich variety of quantum systems provided by the molecular magnetism field strongly motivates the use of SMMs for both quantum information storage and processing purposes.

At the same time, studies in spintronics using magnetic materials in electronic devices have made considerable progress from fundamental studies to practical applications. This technology is based on the discovery of magnetoresistive effects, such as the giant magnetoresistance effect, where metallic spin valves are composed of two metallic layers separated by a non-magnetic one. Depending on the relative magnetization orientation of the two magnets (parallel or antiparallel), a drastic change of the electrical resistance is observed. Nowadays, new directions in spintronics aim at transposing the existing concepts and at developing alternative ones with various types of materials, from inorganic to π-conjugated organic semiconductors [3]. Organic semiconductors are promising since they may offer longer spin relaxation times [4] than conventional transition metals as well as new functionalities (e.g., switchability with light, electric field, *etc*.). In this context, SMMs are interesting candidates to study and preserve quantum coherence of the electronic spin in molecular spintronics devices. Such devices lead the way to the electronic detection and coherent manipulation of SMMs spin states, important for quantum computation schemes at the single molecule level.

In this work, proposed recently [5], we realized a device consisting of SMMs anchored by supramolecular interactions on the sidewall of a chemical vapor deposition (CVD) grown carbon nanotube (CNT), itself connected to a three-terminal (transistor) geometry [6,7]. The CNT acts as a path for conducting electrons so that electronic transport does not occur directly through the magnetic orbitals of the SMM. This prevents charge-induced excitations or relaxation of molecular spin states. In particular, we showed that the electronic transport is extremely sensitive to the orientation of local magnetic moments. This property allows the electrical detection of magnetization reversal of individual molecular spins. In addition, a spin valve effect with two molecules leads to very large variations of the conductance with magnetoresistance ratios of up to several hundred percent. Our approach differs from previous realizations of carbon based spin valves [8–10] and does not imply magnetic leads. Indeed, the spin-dependent transport through this supramolecular spin valve is completely determined by the magnetic properties of the molecular species magnetically coupled to the conducting channel of the CNT. Similar results were recently obtained using graphene nanoconstriction decorated with TbPc_2_ magnetic molecules [11]. In this case, a magnetoconductivity signal as high as 20% was found for the spin reversal. These results show the behavior of multiple-field-effect nanotransistors with sensitivity at the single-molecule level.

The paper is structured as follows. In Section 2, we introduce different methods used to fabricate and measure the supramolecular based junctions. The results section (Section 3) describes the magnetic properties of the pyrene functionalized heteroleptic *bis*-phthalocyaninato-Terbium (III) SMMs (called TbPc_2_ in the following) used in this study. In Section 3.1, we briefly discuss the magnetization reversal mechanisms of the TbPc_2_ SMM, namely QTM and the direct relaxation process, using μ-SQUID measurements on diluted crystals of molecules. Section 3.2 exhibits the electronic transport features of the spin valve, being in the closed quantum dot regime. Then, in Section 3.3, we present the spin-valve behavior revealed by the magneto-conductance measurements under magnetic field sweeps. The anisotropic dependence of the hysteretic conductance jumps is also studied and in good agreement with the expected uniaxial anisotropy of TbPc_2_ SMMs. Finally, Section 4 discusses the spin valve mechanism in further detail and we conclude with a brief outlook.

## 2. Devices Fabrication

In this project we studied about 150 samples of which 28 showed magnetic signals related to the TbPc_2_-SMMs and eight of them were studied in detail, manifesting similar behavior concerning their magneto-conductance. In contrast to our first publication [6], the sample presented in this paper was fabricated using CVD nanotubes. Catalyst islands were designed on SiO_2_ by creating holes by optical lithography in LOR3A resist, which were filled with Fe/Mo catalyst in nanoporous alumina. After liftoff of the resist, nanotubes were grown in a Firstnano CVD oven at 750 °C, whereby methane was used as a carbon source [12]. SWNTs were located by AFM or SEM with respect to lithographically patterned markers and then contacted with 50 nm Pd by standard electron-beam lithography, defining 200 nm long CNT junctions. A solution containing bis(phthalocyaninato)terbium (III) substituted with pyrene groups [13] with a 10^−6^ molarity was dropped on the device and dried under nitrogen flow. Samples with large resistance (>100 kΩ) at room temperature were micro bounded and measurements were carried out in a dilution fridge with a base temperature of 40 mK. The electronic temperature was estimated to be around 150 mK.

## 3. Results

### 3.1. Magnetic Properties of the TbPc_2_ SMMs

**Figure 1:**
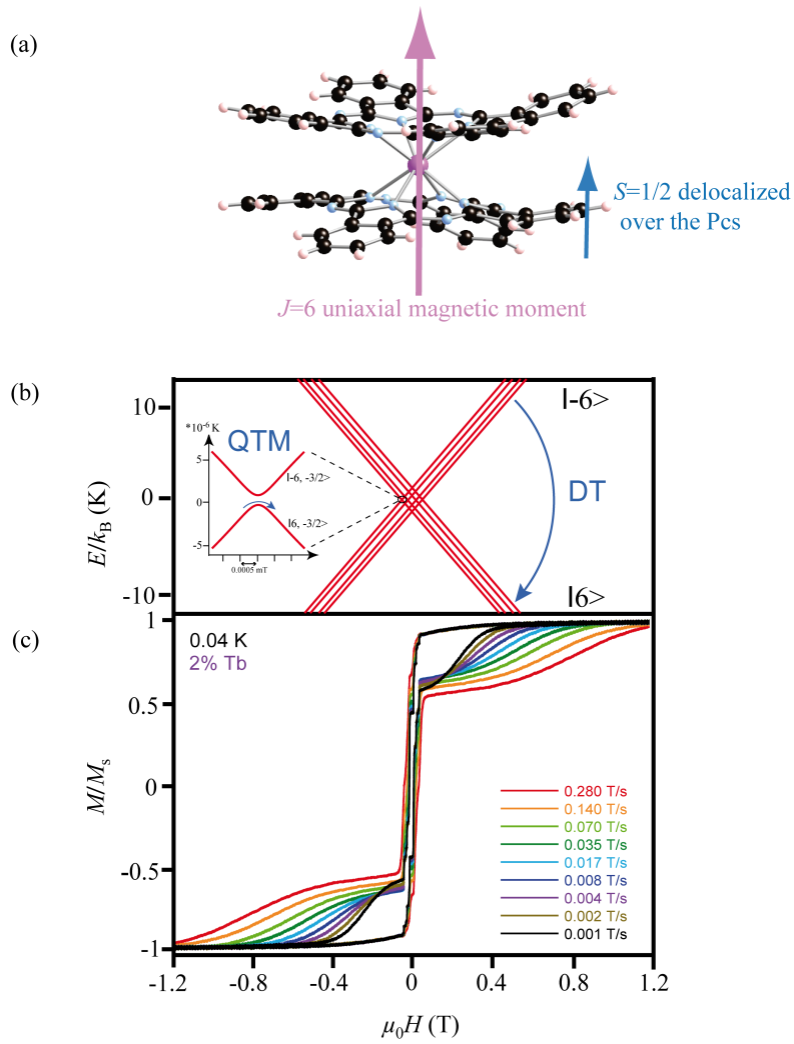
Magnetization reversal mechanisms in the TbPc_2_ SMM (alkyl and pyrene substituents are omitted for reasons of clarity). (**a**) Scheme of the TbPc_2_-SMM. The terbium ion has a *J* = 6 magnetic moment and an unpaired electron is delocalized over the organic part; (**b**) Zeeman diagram calculated for the TbPc_2_ SMM ground state (*J* = 6, |*J*_z_*|* = 6). The interaction with the Tb nucleus spin *I* = 3/2 splits each electronic substate through the hyperfine coupling, providing a path for Quantum Tunneling of Magnetization (QTM) at the anti-crossing of two levels; (**c**) Hysteresis loops of the crystallized TbPc_2_-SMM (2% in the YPc_2_ matrix) measured at 40 mK for different sweeping rates ranging from 1 to 280 mT.s^−1^. QTM reflects in staircase-like steps of the hysteresis loops at low magnetic fields, each step corresponding to a level anti-crossing. Molecules, which did not undergo QTM can relax their magnetization to a lower energy state by the direct transition (DT) occurring at larger magnetic fields.

Among the existing SMMs families, single ion lanthanide complexes are among the most simple and robust systems [13]. Here we focus our attention on the TbPc_2_ SMM based on a single Tb^3+^ ion coordinated to two phthalocyanine (Pc) ligands as depicted in Fdigure 1(a). The SMM behavior originates from the electronic multiplet substructure of the Tb^3+^ ion in its (4f)^8^ electronic configuration leading to a *J* = 6 magnetic moment. The TbPc_2_-SMM exhibits a significantly large axial magnetic anisotropy originating from the strong spin-orbit coupling in lanthanide ions and from the ligand field potential made by the two Pc ligands. It leads to a well-defined ground state configuration (*J* = 6, |*J*_z_*|* = 6) separated from the first excited state (*J* = 6, *J*_z_ = 5) by an energy splitting Δ*E* ~ 600 K [14]. All our measurements were performed at very low temperatures, so that we only consider the lowest energy substates available with *J*_z_ = ± 6, the corresponding Zeeman diagram is plotted in Figure 1(b). Apart from the Terbium(III) ion, the molecule has a spin ½ delocalized over the two phtalocyanines groups [15]. This unpaired electron mediates a magnetic coupling between the Terbium 4f electrons and its environment.

Figure 1(c) presents μ-SQUID magnetization hysteresis loops of the TbPc_2_ SMMs at a temperature of 40 mK. The SMMs have been diluted in an YPc_2_ paramagnetic matrix to minimize any magnetic intermolecular interactions. At an applied magnetic field of −1.2 T along the easy-axis, all magnetic moments in the crystal are saturated along the same direction. Around zero-field, a large number of these magnetic moments switch their magnetization orientation through staircase-like steps of the hysteresis loop. The origin of these magnetization steps is the QTM between the *J*_z_ = 6 and *J*_z_ = −6 substates. Since the other excited substates are, energy-wise, far above the fundamental ones, transitions involving the excited states cannot occur. As a consequence, the origin of QTM in single ion rare earth based SMMs differs strongly from the 3d-metal-cluster SMM cases. This comes from the interaction of the Tb ion with the ligand field. Besides this, the Tb nucleus owns a nuclear spin of *I* = 3/2 with a natural isotopic abundance of 100%. The strong hyperfine coupling in TbPc_2_ leads to a splitting of the *J* = 6 electronic multiplet in several |*J_Z_*>|*I_Z_*> coupled states. These states are visible in the Zeeman diagram in Figure 1(b). QTM can occur through appropriate magnetic field conditions where two such states are brought to resonance at an avoided crossing of two levels (Figure 1(b)). The remaining magnetic moments, which did not undergo magnetization reversal by QTM around the zero magnetic field, start to reverse at about a few hundreds of mT. This transition is visible in the hysteresis loops in Figure 1(c) as a broad field scan rate dependent step. This one phonon mediated mechanism, called the direct transition (DT), is schematized in Figure 1(b).

We highlight that those measurements have been carried out on an assembly of TbPc_2_-SMMs in a crystallized form. One could thus wonder whether the SMM properties may still be observed on sub-monolayers or isolated molecules deposited on a surface. Indeed, SMMs may lose their magnetic properties when attached to metallic surfaces, as was shown e.g., for Mn_12_-acetate [16]. A slight modification of the ions surroundings can lead to a drastic change of the crystal field potential and thus to an alteration of the magnetic properties [17]. However, in the case of the TbPc_2_-SMM, it has been recently demonstrated that the structural and magnetic properties are still conserved [18]. The main fingerprints of SMMs, that is, QTM around zero magnetic field, strong axial anisotropy and high superparamagnetic blocking temperature are, indeed, still present. The TbPc_2_-SMM is one of the most interesting and reliable systems in order to study magnetism at single molecular level. Besides, this molecule is very well suited to be attached to sp^2^ carbon nanomaterials, such as carbon nanotubes or grapheme [19] via supramolecular π-π interactions. This strategy has been used to build our supramolecular spin valve and is described in the following.

### 3.2. Electronic Transport through Carbon Nanotubes Functionalized by TbPc_2_-SMMs

**Figure 2:**
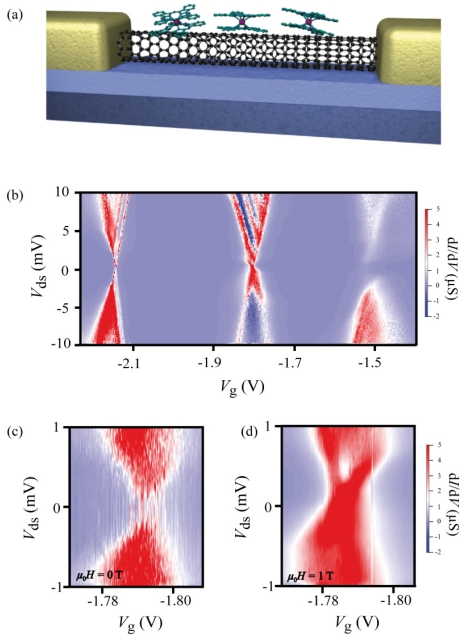
Electronic transport in carbon nanotube quantum dots with grafted TbPc_2_ molecules. (**a**) Artist view of the device scheme, consisting of an electrically connected carbon nanotube junction, laterally coupled to isolated TbPc_2_-SMMs; (**b**) Color-scale plots of the differential conductance *dI/dV* at temperature *T* = 40 mK, as a function of source-drain voltage *V_sd_* and back-gate voltage *V_g_*, displaying the charge stability diagram in the Coulomb blockade regime. The typical charging energy is about 20 meV; (**c–d**) Enlarged views of (b), showing the charge degeneracy point around *V_g_* = 1.79 V at constant static magnetic fields (c) *μ*_0_*H* = 0 T and (d) *μ*_0_*H* = 1 T.

The original geometry of our devices is presented in Figure 2(a). A CNT, contacted with non-magnetic electrodes, forms a quantum dot (QD), which is laterally coupled to several TbPc_2_-SMMs through π-π stacking interaction. Indeed, phtalocyanine groups can be functionalized with pyrenes ligands, allowing a supra-molecular anchoring point and a better coupling to π-conjugated systems such as carbon nanotubes [20].

When deposited, the TbPc_2_-SMM anchors on the nanotube in a way that the substituted Pc ligand of the molecule comes directly in contact with the nanotube surface. The energy gain through supramolecular interactions is maximized by the formation of strong aromatic π-π and C-H-π stacking interactions between the substituted Pc and CNT. Because of the efficient hybridization between Pc and CNT orbitals [21], we can assume that the unpaired electron delocalized over the Pc ligands has strong interaction with the conduction electron of the nanotube.

The supra-molecular device structure is cooled down in a ^3^He/^4^He dilution refrigerator to 40 mK and a differential conductance measurement is conducted with an Adwin real-time acquisition system, programmed in a lock-in mode. The lock-in amplitude and frequency are set to 50 μV and 33 Hz, respectively. The QD characteristics are measured by bias spectroscopy, that is, the differential conductance d*I*/d*V* is plotted in a color code as a function of the back-gate and bias voltages. Figure 2(b) shows the standard Coulomb diamond expected for a CNT QD with a charging energy around 20 meV. Figures 2(c,d) display a zoom on the degeneracy points without and under magnetic field (1 T). At low bias (<1 mV) and without magnetic field (Figure 2(c)), the degeneracy point has a noisy conductance, which fluctuates between two values. Then under 1 T, the conductance becomes stable and closed (Figure 2(d)). These features reveal the presence of an extra tunnel barrier in the QD [22], which can be modulated by the magnetic field.

### 3.3. Characterization of the Strongly Anisotropic Spin-Valve Effect

**Figure 3:**
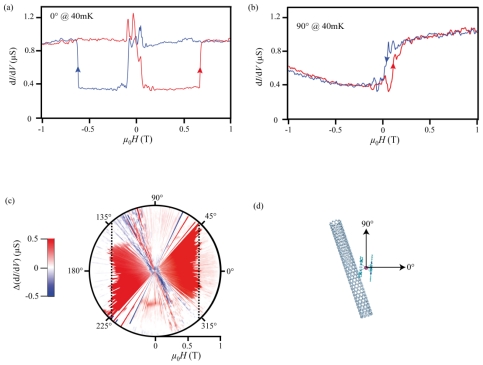
Conductance hysteresis loops of the supramolecular spin valve. (**a–b**) Differential conductance *dI/dV* measured at *T* = 40 mK as a function of in-plane magnetic field *μ*_0_*H* applied respectively along (**a**) the easy axis direction (0°); and (**b**) the hard direction (90°) of magnetization. The red and blue arrows indicate the magnetic field sweep directions; (**c**) Color-scale plot of the *dI/dV* hysteresis (obtained from the difference between both field sweep directions) as a function of the applied magnetic field angle. The white color code is associated to zero hysteresis (reproducible *dI/dV* curves); (**d**) Relative disposition of the molecule with respect to the nanotube. The magnetic hard axis is 30° tilted from the nanotube axis.

The magnetoresistance measurements, at the previously mentioned degeneracy point (see Figure 2), are presented in Figure 3(a). At −1 T, the differential conductance is saturated to its maximum value. Sweeping up the magnetic field at 20 mT/s until zero-field, d*I*/d*V* drops down abruptly to its minimum value. When still increasing the field, d*I*/d*V* abruptly recovers its original value. The complete measurement from −1 T to +1 T (trace) and back to −1 T (retrace) forms a hysteresis loop, which is characteristic of a spin-valve device.

Another remarkable feature of our device is its anisotropic response: we observed that the field values at which sharp conductance jumps are measured, called switching fields, depend strongly on the direction of the applied field. From a certain angle, our magnetic field is not strong enough to observe switching, and the hysteresis disappears (Figure 3(b)). In this case, the differential conductance does not depend on the field history; d*I*/d*V* is minimum for negative magnetic field and maximum for positive magnetic field, demonstrating that the switching near zero-field occurs independently from the one taking place at larger field. Thereby, we have to consider several independent magnetic objects to explain the data.

In order to go one step further, we have plotted on Figure 3(c) the hysteresis amplitude (difference between trace and retrace) as a function of the applied field direction. It turns out that the switching field occurring at high value (the dashed line on Figure 3(c)) describes a straight line in the field space. The projection of those points along one axis stays constant, which is the fingerprint of the Ising like uniaxial anisotropy of the TbPc_2_-SMM family.

**Figure 4:**
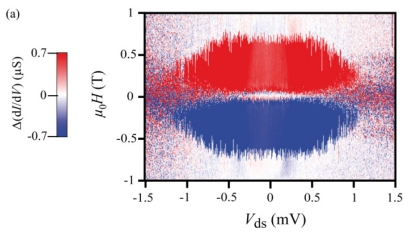

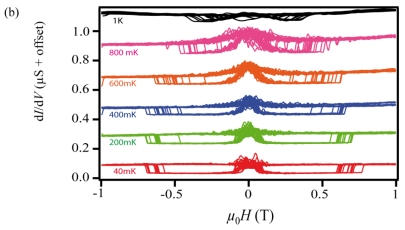
Bias and temperature dependences of the conductance hysteresis loops: (**a**) Color scale map of the *dI/dV* hysteresis as a function of in-plane magnetic field and source drain voltage *V_ds_*. The magnetic hysteresis are suppressed above *V_ds_* = ± 1 mV; (**b**) 15 records of conductance hysteresis loops for several temperatures ranging from 0.04 to 1 K at a constant sweep rate of 50 mT/s. The curves for *T* > 40 mK are offset by a multiple of 200 nS for clarity.

Figure 4(a) shows the dependence of the hysteresis on the bias voltage applied to the quantum dot. This bias dependence shows that the hysteresis persists until 1 mV, but above this value, any residual hysteresis is smaller than the noise level. Furthermore, this measurement provides additional information: the switching field evolves with the bias voltage. We conclude from this observation that the conduction electrons can excite the molecules for larger bias voltages. Indeed, the energy of the conduction electrons increases with the bias voltage. A part of this energy might be transferred to the molecule via the electron density on the Pc-ligands and therefore to the anisotropic Tb spin system.

As the temperature increases, the amplitude of the magnetoresistance decreases (Figure 4(b)). Indeed the spin-phonon interaction increases with the temperature and affect the spin-coherence. The magnetoresistance feature is lost around 1 K, which is below the TbPc_2_-SMMs blocking temperature but is in agreement with an exchange interaction of a few hundreds of μV. The blocking temperature can be roughly estimated to be around 1.5 K, in agreement with recent XMCD measurements of TbPc_2_-SMMs monolayers [18].

## 4. Discussion

The spin-valve features can be explained by a simple model involving two distinct molecules: one with a close to one QTM probability and another one only subjected to DT. Under a high magnetic field, the magnetic moments of both molecules are polarized in parallel. This situation corresponds to the high conductance regime. When the magnetic field is reduced, one of the molecule experiences QTM close to zero field, whereas the other stays in the same state. The device is then in an antiparallel configuration and the conductance is minimum. Finally, when the following switching field is reached, meaning when the second molecule undergoes a DT, the parallel arrangement, and therefore the original conductance value, are recovered. This is, of course, the idealized case. In fact, by looking closely at the QTM region, we can observe several abrupt changes of conductance. It means that several molecules (more than two) are involved in the close to zero field process. However, only one molecule relaxes at larger fields via a DT.

**Figure 5:**
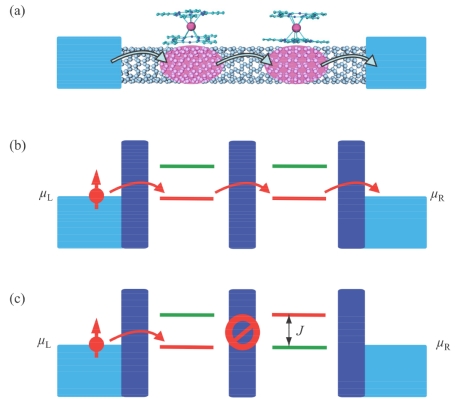
(**a**) Scheme of the localized dots induced by hybridization between the molecules and the nanotube; (**b**) Both molecules are polarized in the same manner. It induces a Zeeman splitting identical for both sets of localized states; (**c**) In the antiparallel configuration the Zeeman splitting is inhomogeneous, preventing spin transport through the device, unless a bias higher than the exchange interaction is applied.

From a microscopic point of view, each of these molecules interacts with the nanotube by creating a localized spin-polarized state in its vicinity (schematized in Figure 5(a)). A dipolar interaction is not sufficient to explain such an effect. Indeed, the dipolar interaction between the *S* = 1/2 radical on the Pcs and the conduction electrons cannot exceed a few tens of mT (1/2 *μ*_B_ creates a 1/2 T dipolar field at 1 Å, and ~20 mT at 3 Å). Also, even if we consider the effect of the moment *J* = 6 on the conduction electron, the dipolar field is of the same order of magnitude, which is too small to explain the strength of the effect. Considering an exchange interaction, mediated by the π-electron density in the organic Pc ligand, seems to be more realistic. Indeed, Hu *et al*. [23] have shown that spin-polarization may occur through the interaction between a π-system delocalized over a carbon chain and latterly coupled spin radical. As shown by Gambardella *et al*. [24] in a similar system, the electron density on the Pc ligand is able to mediate the magnetic information by a strong exchange interaction. The molecules induce localized states on the nanotube by lifting the spin degeneracy through this interaction (Figure 5(b)). The strength of this interaction can be estimated to be around 200 μV by looking at the gapped degeneracy point in Figure 2(d). The spin level splitting is inhomogeneous along the tube when both molecules are in the antiparallel configuration, as a result of the mismatch between energy levels for a same spin (Figure 5(c)). This barrier can be overcome by applying a bias voltage higher than the exchange interaction between the molecule and the conduction electrons.

## 5. Conclusions

We have reported on the characterization of a fully molecular spin-valve made of a carbon nanotube laterally coupled to a few single-molecule magnets determining for the first time the strength of the exchange energy between the SMMs. The interaction between both objects is strong enough to allow an abrupt modification of the conductance by changing the magnetization direction of only one molecule. As a result, the device exhibits a spin-valve effect and the magneto-conductance ratio can reach a few hundreds of percents. These features are obviously related to the grafted TbPc2-SMMs. Indeed, the anisotropic response clearly corresponds to an Ising-like uniaxial magnetic system. Our results open a pathway toward the fabrication of an all-organic spintronic device by the use of supramolecular self-organization techniques. Moreover, the high sensitivity of the device allows the characterization and the control of a single localized spin. Thus, an entanglement between different spin systems could also be possible using the CNT as tunable bus.
